# Effects of a High Protein Food Supplement on Physical Activity, Motor Performance and Health Related Quality of Life of HIV Infected Botswana Children on Anti-Retroviral Therapy (ART)

**DOI:** 10.3934/publichealth.2017.3.258

**Published:** 2017-05-26

**Authors:** Leapetswe Malete, Lucky Mokgatlhe, Maria Nnyepi, Jose Jackson, Fujun Wen, Maurice Bennink, Gabriel Anabwani, Jerry Makhanda, Ibou Thior, Philemon Lyoka, Lorraine Weatherspoon

**Affiliations:** 1 Michigan State University, East Lansing, Michigan, USA; 2 University of Botswana, Gaborone, Botswana; 3 Botswana-Baylor Children's Clinical Centre of Excellence, Gaborone, Botswana; 4 Baylor College of Medicine, Pediatric Retrovirology, Houston, Texas, USA; 5 Botswana Harvard Partnership, Gaborone, Botswana; 6 University of Fort Hare, Alice, South Africa

**Keywords:** physical activity, motor performance, health related quality of life, ART

## Abstract

**Objective:**

Despite existing evidence about the benefits of nutrition, physical activity (PA) and sport to the overall health and wellbeing of children, knowledge gaps remain on this relationship in children living with chronic conditions like HIV/AIDS. Such knowledge should inform context specific programs that could enhance the quality of life of children. The purpose of this study was to examine the effects of integrating a nutrition intervention (culturally tailored food supplement) into antiretroviral therapy (ART) on psychosocial outcomes and physical activity among HIV-positive children in Botswana.

**Method:**

201 HIV-positive children (6–15 years; M = 9.44, SD = 2.40) were recruited and randomly assigned (stratified by age and gender) to two groups. The intervention group (n = 97) received a high protein (bean-sorghum plus micronutrients) food supplement, while the control group (n = 104) received a sorghum plus micronutrients supplement. Participants were followed over 12 months. Anthropometric measures, PA, motor performance, and health related quality of life (HRQL) were collected at baseline, 6 and 12 months.

**Results:**

Mixed repeated-measures ANOVA revealed a significant time effect of the food supplement on target variables except body fat percentage, speed, and school functioning. Time × treatment interaction was found for physical functioning, psychosocial functioning and total quality of life score. Scores on physical functioning and total of quality life in the intervention group significantly increased from baseline to 6 months compared with the control group (*p* = 0.015).

**Conclusion:**

A combination of ART and nutritional intervention had a positive effect on physical functioning and total quality of life of HIV-positive children in this study. There were also improvements to physical activity and motor performance tests over time. More research is needed on long term effects of nutrition and PA interventions on HRQL in children living with HIV.

## 1. Introduction

Evidence-based interventions aimed at the management, care as well as reductions in the spread of the HIV virus and its impact on communities have paid huge dividends. Key to these interventions is the effort to increase the national coverage of antiretroviral therapy (ART) to all infected individuals. The latest UNAIDS report covering 160 countries shows that, the number of people who enrolled into ART programs have more than doubled. Also AIDS related deaths have declined by 36% between 2010 and 2015 [1]. Over the 5-year period, the global ART coverage reached 46% but for East and Southern Africa it increased from 24% to 54%. This is significant to the socio-economic development of these regions and the wellbeing of their predominantly youthful populations. This global health success story has, however, ushered in new challenges, mostly health related quality of life (HRQL) of people on ART.

The risks factors associated with ART such as drug resistance, toxicity and metabolic syndrome adversely impact HRQL and increase the risk of cardiovascular disease among people living with HIV [2]–[4]. While these cardiovascular risk factors are not uncommon in the general population, their high prevalence among HIV infected individuals present an important challenge and require urgent intervention. Diet and physical activity (PA) may be key strategies to reducing the incidence of cardiovascular disease and enhancing the quality of life within the general population [3],[5]. There is evidence that acute exercise could reduce the waist-to-hip ratio, visceral fat, and levels of cholesterol, triglyceride and LDL-c, thereby reduce the adverse metabolic effects associated with ART [3],[4],[6].

If good nutrition and moderate to vigorous PA are associated with good health outcomes in the general population they may be equally effective among individuals living with HIV and other chronic conditions. However, there are significant knowledge gaps on the impact of nutrition and PA and their relationship to the health and wellbeing of children on ART. Such evidence, especially from the regions of the world that have a significant number of children and adolescence on ART will be key to the design of intervention strategies that combine nutrition, PA for the care and management of HIV. This will be critical to the quality of life for many generations that have to live with the disease. Further, diet and physical activity have cultural and other contextual nuances that are important to know in order to develop sustainable and effective interventions. The existing literature on HRQL in children living with chronic conditions demonstrates the value of investigating these factors but there is a limited number of studies on children living with HIV and African populations in particular [7],[8].

Therefore, this paper presents findings on the effects of a commonly consumed food product (sorghum) that is protein-micronutrient enhanced compared to one that is enhanced with micronutrients only on the HRQL, physical activity and motor performance of children on ART over time. These changes were considered an important proxy of children's wellbeing associated with the intervention. Here, PA, motor performance and HRQL are considered as representing important proxies of children's wellbeing. These findings are part of a larger randomized controlled trail published elsewhere [9],[10] on changes to nutritional status, PA, cognitive and immune functioning in children receiving ARV and a nutritional intervention over 12 months in Botswana.

## 2. Materials and Methods

### 2.1. Participants and Procedure

201 HIV-positive children aged six to 15 years were randomly recruited at ART treatment baseline from Botswana Baylor College of Medicine Children's Clinical Center of Excellence (BBCCCE) in Gaborone. Participants met the eligibility criteria for inclusion: aged 6–15 years, CD4 cell count < 700/mm^2^, on ART, and no documented records of receiving a nutritionally enhanced food product with added energy and/or micronutrients within six months prior to the study. The randomization was achieved by utilizing a random number table. Ninety-seven participants were randomly assigned to the intervention group compared to 104 participants in the control group. The duration of the intervention was 12 months. During this period, participants' BMI percentile, body fat percentage, physical activity, basal metabolic rate, motor performance and HRQL were measured at three points: baseline, 6 months and 12 months.

The intervention study was conducted through a partnership including Michigan State University, University of Botswana, Botswana-Harvard Partnership, Ministry of Health of Botswana, Botswana Baylor College of Medicine Children's Clinical Center of Excellence (BBCCCE), and Foods Botswana, a local milling company. Therefore, the approvals from the Institutional Review Board were obtained from Michigan State University, University of Botswana, and Ministry of Health of Botswana. Written and verbal consent were obtained from the parents/legal guardians. The children were also asked to provide verbal assent prior to data collection.

### 2.2. Measures

Each participant completed a demographic questionnaire that included age, gender, and household income. Anthropometric variables, including height, weight, body fat percentage, were measured. Height was measured without shoes using a portable scale (Seca, Hamburg, Germany) to the nearest 0.1 cm. Weight was measured in light clothes using a calibrated digital scale (Tanita, Tokyo, Japan) to the nearest 0.1 kg. BMI was calculated as weight (kg)/Height (m^2^). Body fat percentage was measured using a Tanita TBF 300 bioelectrical impedance scale (Tanita Corporation of America, Inc., IL, and USA). Testing was done under a state of self-reported normal hydration and no strenuous exercise prior to testing.

Motor performance was measured using the Children Motor Performance Test (CMPT), which was adapted from the short-form of the Bruininks-Oseretsky Test of Motor Proficiency (BOTMP-SF) [11]. It has been demonstrated that the BOTMP-SF is appropriate for use among children aged 4 to 21 years [12]–[14] and good for assessment of general motor functioning [15],[16]. The adapted CMPT test comprised of five areas: speed, power, upper power and coordination, grip strength and cardiovascular endurance.

Physical activity was measured using the Habitual Physical Activity Questionnaire (HPAQ), which was an abridged version of the Physical Activity Questionnaire (PAQ-C) and the short form of the International Physical Activity Questionnaire (IPAQ-SF). The 10-item PAQ-C has been shown to have good reliability and validity among the children aged 8 to 16 years [17],[18]. It has been reported to have a reliability of about 0.65 and a validity of 0.3 (95% CI 0.23–0.36) compared to objectively measured physical activity with ACTi Graph accelerometers among a large population drawn from across 12 countries [19]. The adapted HPAQ questionnaire was used to evaluate children's physical activity and sedentary behaviors including walking, playing, bicycling, and home chores such as carrying light or heavy weights as well as time resting or lying down over the past 7 days. The following formulas were used to calculate the weekly energy expenditure: Low to Moderate MET-minutes/week = 3.0 × walking/playing minutes × walking/playing days and vigorous MET-minutes/week = 5.0 × vigorous activity minutes × vigorous days [20].

The HRQL was assessed using the revised Pediatric Quality of Life Inventory 4.0 (PedsQL 4.0) [21]. Previous studies have demonstrated that PedsQL 4.0 has good reliability and validity across diverse populations and settings [21]–[25]. The revised 23-item inventory assessed four dimensions: (1) physical functioning (eight items); (2) emotional functioning (five items); (3) social functioning (five items); and (4) school functioning (five items). We used the same procedure of scaling and scoring used in the PedsQL 4.0 and reported elsewhere [21],[24]. The Cronbach's alphas for the four HRQL factors ranged from 0.69 to 0.84, which shows that the inventory has good internal consistency and is suitable for use with the current sample.

### 2.3. The Intervention

Participants were enrolled in the study at the start of the ART program offered by the BBCCCE. Over the 12 months, the experimental group received a high protein (sorghum-bean) micronutrient fortified food supplement while the control group was given sorghum only micro-nutrients fortified product. The children refilled their ART medication every three months. Clinical tests that included viral load, CD4 cell count and CD4 percentage were also done every three months. The food products were distributed on monthly basis on the days the children visited the clinic for health checks. Each child was given two bags each weighing 5 kg. This was estimated to be sufficient for daily consumption by a child in a family of four, which was the average household in Botswana. The estimate was also based on typical household use of sorghum porridge in Botswana diets. Parents and children were instructed to use the fortified sorghum instead of the non-fortified one at least once a day. The actual amount of fortified sorghum porridge consumed by the children was established through follow up interviews to parents and children in the study. Monthly feedback and results of a nutritional quality and acceptability study of the two products showed that they were heavily consumed and had higher nutrition and sensory attributes compared to the regular sorghum product [10].

### 2.4. Statistical Analysis

Descriptive statistics, such as mean and standard deviation, were used to present the participants' characteristics. Baseline differences between intervention and control group were examined by conducting independent samples t-tests. Missing data were handled by employing expectation maximization (EM), which has been demonstrated to be a superior estimate at different levels of missing data compared with mean substitution [26]–[28]. Repeated measures ANOVAs were done to compare the change in BMI, body fat percentage, the levels of physical activity, motor performance, and how these relate to health-related quality of life of both the intervention and control groups from baseline to 6-months and12-months. All effect sizes were calculated by using Cohen's d = (M_1_ − M_2_)/σ_pooled_, and values of 0.2, 0.5 and 0.8 represented small, moderate and large effects, respectively. All data analyses were performed with SPSS (SPSS Inc., Chicago, IL) and an alpha level of 0.05.

## 3. Results

### 3.1. Preliminary Analyses

A total of 201 participants aged 6–15 years were recruited to participate in this study. Ninety-seven participants (M = 9.29 years; SD = 2.49) were randomly assigned to the intervention group and 104 (M = 9.57 years; SD = 2.34) to the control group. Approximately 44% (n = 43) of the participants in the intervention group were female while the control group was 43% (n = 45) female. Most of the children came from predominantly low-income backgrounds. About 72.9% of the parents/caregivers reported having no running water in their houses and using tap water from a stand-pipe in the yard, and 42.4% had a monthly income of less than P600 (US $110). A significant proportion of the children in both groups were stunted (42.9%), underweight (27.4%) and wasted (5.1%).

Independent samples t-tests were run to test for group differences in the study variables at baseline. Results showed no significant differences in BMI, body fat percentage, physical activity level, motor performance, physical functioning, emotional functioning, school functioning, and psychosocial functioning at baseline between the intervention and control groups. However the control group (M = 96.83, SD = 6.39) had significantly higher scores on social functioning than the experimental group (M = 93.51, SD = 13.82, *p* = 0.032) and total score of HRQL (M= 86.70, SD=7.16) compared to the experimental group (M = 83.87, SD = 10.48, *p* = 0.031) at baseline. This suggests that overall the two groups were comparable at baseline. A summary of demographic and baseline PA and motor performance data is provided in Table 1. Table 2 presents baseline summary data for HRQL for the two groups.

Next we ran repeated measures ANOVAs to examine changes in study variables for both the intervention and control groups from baseline to 6 months and 12 months. These results are summarized in Table 3.

**Table 1.** Demographic and Baseline Data for the Intervention and Control Group.

Variable	Intervention (n = 97)	Control (n = 104)	*p* value ^a^
	M ± SD or n (%)	M ± SD or n (%)	
Age	9.29 ± 2.49	9.57 ± 2.34	0.415
Female	43 (44.3%)	45 (43.3%)	0.529
BMI	15.22 ± 1.46	15.38 ± 1.61	0.486
BFP	12.76 ± 5.98	12.51 ± 5.47	0.758
**Physical activity**			
LMPA	4.05 ± 3.27	3.79 ± 2.67	0.529
VPA	2.17 ± 2.84	2.52 ± 2.62	0.365
**Motor performance**			
Speed (seconds/20 m)	5.54 ± 1.63	5.21 ± 1.37	0.118
Lower power (m)	0.99 ± 0.32	1.02 ± 0.30	0.572
UPC	15.61 ± 6.29	16.44 ± 6.10	0.341
Grip strength	9.95 ± 4.09	10.73 ± 4.07	0.175
Cardiovascular endurance	2.26 ± 1.19	2.49 ± 1.24	0.185

NB: BMI = body mass index; BFP = body fat percentage; LMPA = low to moderate physical activity (KMets/week); VPA = vigorous physical activity (KMets/week); UPC = upper power and coordination (m).

**^a^** Independent samples t-test.

**Table 2.** Health Related Quality of Life at Baseline for the Two Groups.

HRQL	Intervention (n = 97)	Control (n = 104)	*p* value ^a^
Physical functioning	88.60 ± 14.34	92.14 ± 11.66	0.056
Emotional functioning	87.41 ± 14.43	88.80 ± 12.14	0.460
Social functioning	93.51 ± 13.82	96.83 ± 6.39	0.032 *
School functioning	66.34 ± 15.93	69.01 ± 14.93	0.220
Psychosocial functioning	82.42 ± 10.96	84.89 ± 7.55	0.067
Total score of HRQL	83.87 ± 10.48	86.70 ± 7.16	0.031 *

HRQL = Health related quality of life.

**^a^** Independent samples t-test.

* *p* < 0.05.

**Table 3.** Intervention Effects over Time.

Variables	Baseline	6-month	12-month	Time × treatment effect (*p*-value)		
	Treatment	Treatment	Treatment	Baseline to 6-month	6-month to 12-month	Baseline to 12-month
	Control	Control	Control			
BMI	15.22 (1.46)	15.18 (1.46)	15.50 (2.56)	0.337	0.465	0.836
	15.38 (1.61)	15.44 (1.59)	15.59 (1.65) **			
BFP	12.76 (5.98)	12.78 (6.12)	12.17(6.01)	0.532	0.796	0.690
	12.51 (5.47)	12.87 (5.26)	12.19 (4.98)			
LMPA(KMets/Week)	4.05 (3.27)	3.60 (1.81)	3.37 (1.42)	0.831	0.056	0.352
	3.79 (2.67)	3.24 (1.50)	3.56 (1.39)			
VPA(KMets/Week)	2.17 (2.84)	2.47 (2.17)	3.20 (1.87) **	0.127	0.577	0.263
	2.52 (2.62)	2.15 (1.74)	3.07 (1.76) **			
Speed (seconds/20m)	5.54 (1.63)	5.27 (1.17)	5.43 (1.29)	0.286	0.343	0.706
	5.21 (1.37)	5.19 (1.28)	5.16 (0.99)			
Lower power (m)	0.99 (0.32)	1.01 (0.32)	1.08 (0.30) *	0.860	0.852	0.743
	1.02 (0.30)	1.03 (0.31)	1.09 (0.26) *			
UPC	15.61 (6.29)	16.14 (6.05)	16.89 (6.05) *	0.220	0.735	0.140
	16.44 (6.10)	16.25 (6.30)	16.79 (6.02)			
Grip strength	9.95 (4.09)	11.44 (4.79) **	12.84 (5.40)**	0.508	0.953	0.633
	10.73 (4.07)	12.03 (4.58) **	13.38 (5.02) **			
Cardiovascular Endurance	2.26 (1.19)	2.26 (1.10)	2.40 (1.18)	0.583	0.761	0.899
	2.49 (1.24)	2.39 (1.25)	2.59 (1.10)			
Physical functioning	88.60 (14.34)	95.13 (8.32)**	93.95 (9.95)**	0.015 *	0.066	0.177
	92.14 (11.66)	94.04 (7.80)	95.11 (8.32) *			
Emotional functioning	87.41 (14.43)	91.88 (12.24) *	91.54 (11.95) *	0.108	0.370	0.444
	88.80 (12.14)	89.62 (12.38)	91.28 (13.60)			
Social functioning	93.51 (13.82)	97.21 (9.50) *	95.83 (10.01) *	0.087	0.124	0.668
	96.83 (6.39)	97.48 (7.84)	98.52 (5.71)			
School functioning	66.34 (15.93)	70.16 (17.70)	66.96 (16.62)	0.393	0.403	0.932
	69.01 (14.93)	70.34 (18.04)	69.45 (16.11)			
Psychosocial functioning	82.42 (10.96)	86.42 (8.33) **	84.78 (8.56)	**0.052**	0.077	0.523
	84.89 (7.55)	85.81 (8.46)	86.42 (6.41)			
Total score of HRQL	83.87 (10.48)	88.59 (6.61) **	87.07 (7.62) *	0.016 *	0.027 *	0.316
	86.70 (7.16)	87.87 (7.06)	88.59 (5.42) *			

NB: BMI = body mass index; BFP = body fat percentage; LMPA = low to moderate physical activity; VPA = vigorous physical activity; UPC = upper power and coordination (m); HRQL = Health related quality of life. ** *p* < 0.01; * *p* < 0.05.

### 3.2. Physical Activity

#### 3.2.1. Low to Moderate Physical Activity (LMPA)

There was a near significant time × treatment interaction for LMPA from 6 months to 12 months (F = 3.70, *p* = 0.056, d = −0.271). Though not statistically significant, some modest increases in LMPA were observed in the control group (See Table 3) whereas no LMPA increases were observed in the intervention group from 6 months to 12 months. There were no significant main effects for time and treatment for both groups.

#### 3.2.2. Vigorous Physical Activity (VPA)

**Figure 1.**
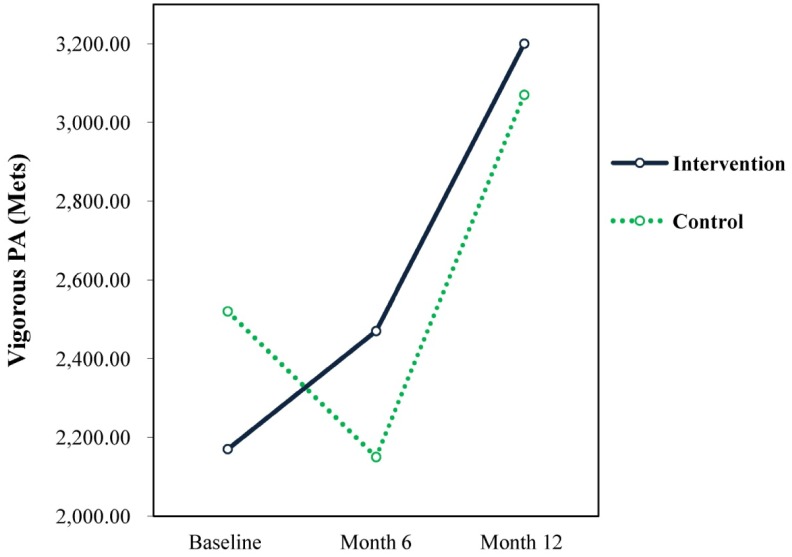
Vigorous PA.

There was a significant increase in VPA from baseline and 6 months to 12 months for both groups. No significant time × treatment interactions effects were found. Results also showed a significant increase in VPA from baseline to 12 months for the intervention group (F = 9.44, *p* = 0.003, d = 0.448) and from 6 months to 12 months (F = 15.00, *p* = 0.001, d = 0.526) for the control group (see Figure 1). The intervention group had a slightly lower score than the control group at baseline. The group's scores increased significantly above that of the control group by the sixth month and remained higher at 12 months (see Figure 1).

### 3.3. Motor Performance

Results showed no significant time × treatment interaction effects or main effects for treatment for any of the motor performance measures across the 12 months of the intervention. There were however significant main effects for time for low body power, upper power and coordination, and grip strength. Lower body power significantly increased for both groups from baseline (M = 1.01, SD = 0.30) to 12 months (M = 1.09, SD = 0.26; F = 23.26, *p* = 0.001, d = 0.272). However, the changes in lower body power from baseline to 6 months were not significant for both groups.

**Figure 2.**
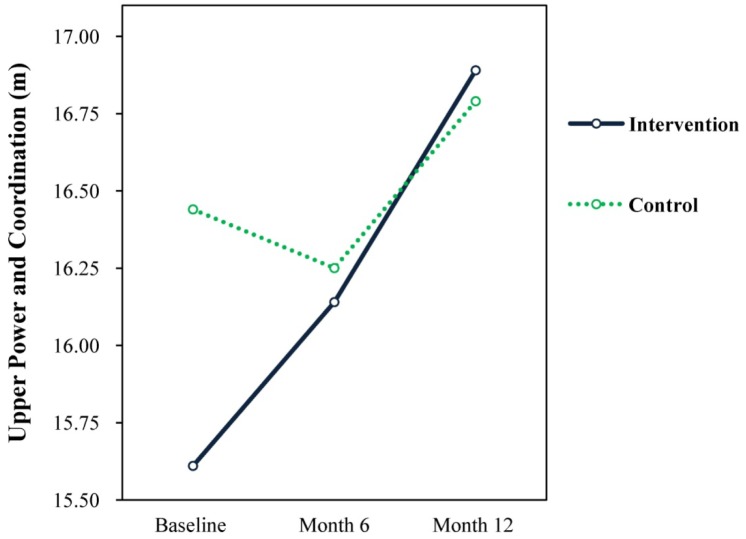
Upper power and coordination.

**Figure 3.**
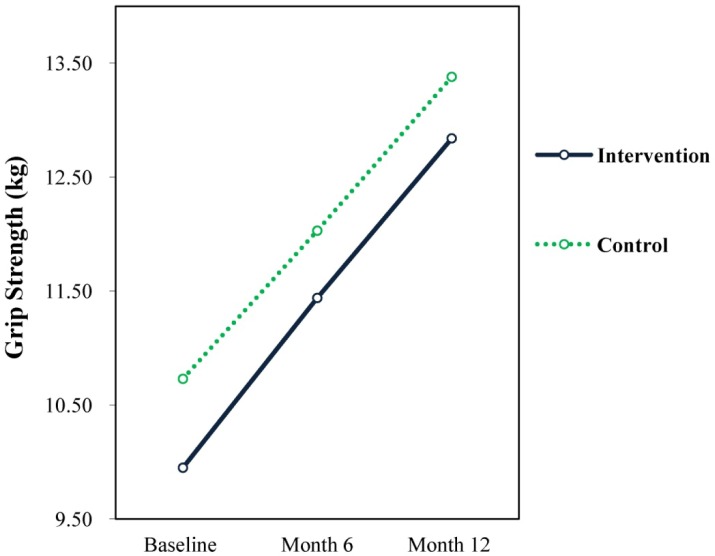
Grip strength.

For upper power and coordination, there were significant main effects for time (F = 4.16, *p* = 0.016, d = 0.130) from baseline (M = 16.05, SD = 6.20) to 12 months= 16.84, SD = 6.02) for both groups. There were no significant changes from baseline to 6 months. There were significant increases in grip strength for both groups over time indicating that grip strength irrespective of group improved from baseline (M = 10.35, SD = 4.08) to 6 months (M = 11.74, SD = 4.68; F = 94.82, *p* = 0.001, d = 0.317) and from baseline (M = 10.35, SD = 4.08) to 12 months (M = 13.12, SD = 5.20; F = 178.41, *p* = 0.001, d = 0.593), as well as from 6 months (M = 11.74, SD = 4.68) to 12 months (M = 13.12, SD = 5.20; F = 50.83, *p* = 0.001, d = 0.279). The changes to upper power and coordination and grip strength are illustrated in Figures 2 and 3.

### 3.4. BMI and Body Fat Percentage

Changes to BMI and body fat percentage are considered important indicators of progress in the children's wellbeing and capacity to engage in PA and other daily activities. Therefore we examined the relative changes to these variables by group over time. Results from the ANOVA revealed no significant time × treatment interactions for BMI from baseline to 6 months and 12 months. However, there were significant time effects, indicating an increase in BMI for both the treatment and control groups from baseline (M = 15.30, SD =1.54) to 12 months (M = 15.55, SD = 2.13; F = 4.51, *p* = 0.032, d = 0.135), 6 months (M = 15.31, SD = 1.53) to 12-months (M = 15.55, SD = 2.13; F = 4.53, *p* = 0.043, d = 0.129), but not from baseline (M = 15.30, SD = 1.54) to 6 months (M = 15.31, SD = 1.53). Results also showed that from baseline (M = 15.37, SD = 1.61) to 12-months (M = 15.59, SD = 1.65) there was a significant change in BMI (F = 8.14, *p* = 0.005, d = 0.135) but only for the control group.

**Figure 4.**
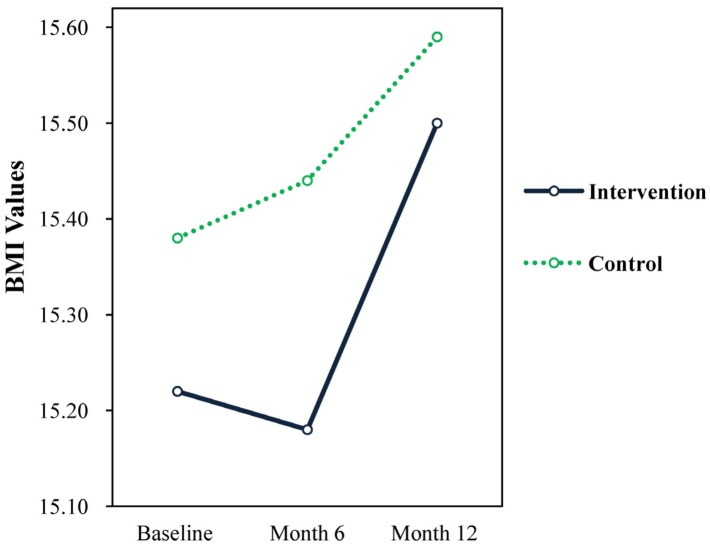
BMI.

**Figure 5.**
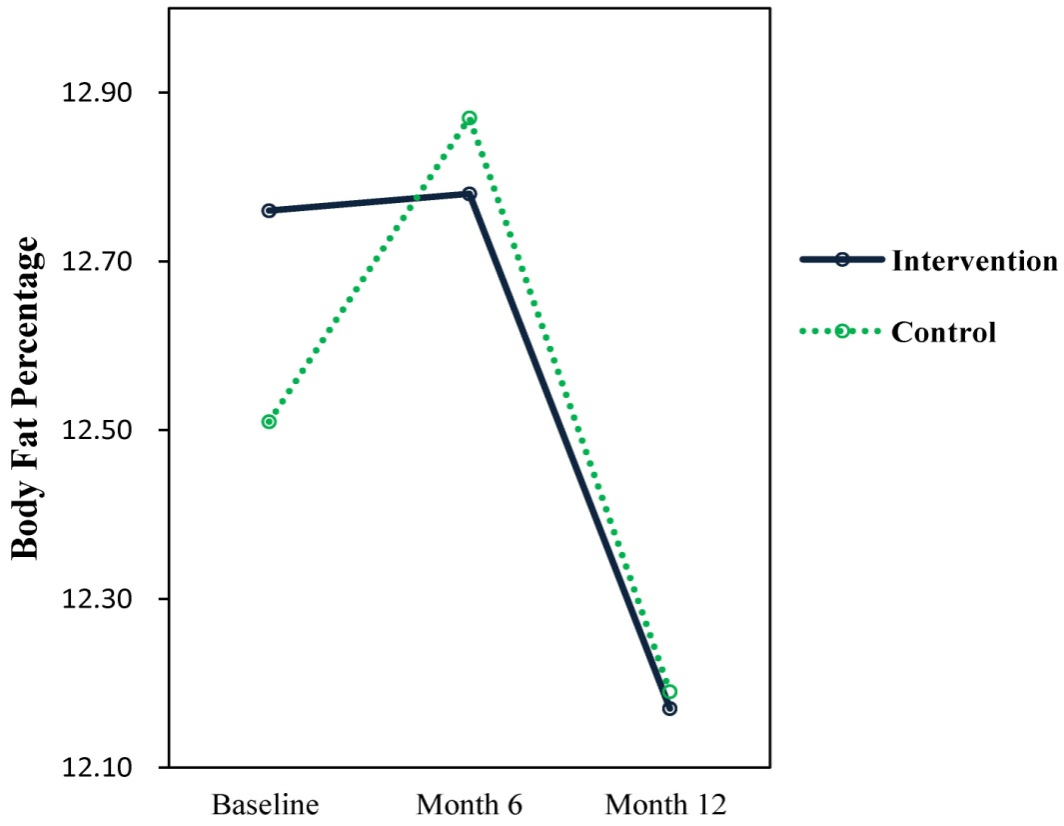
Body Fat percentage.

For the body fat percentage, results showed that there were no significant time × treatment interaction effects, no main effects for time or treatment for both groups. It is worth noting that the children in both control and treatment were underweight. Changes to BMI and body fat percentage are presented in Figure 4 and 5.

### 3.5. Health Related Quality of Life

#### 3.5.1. Physical Functioning

**Figure 6.**
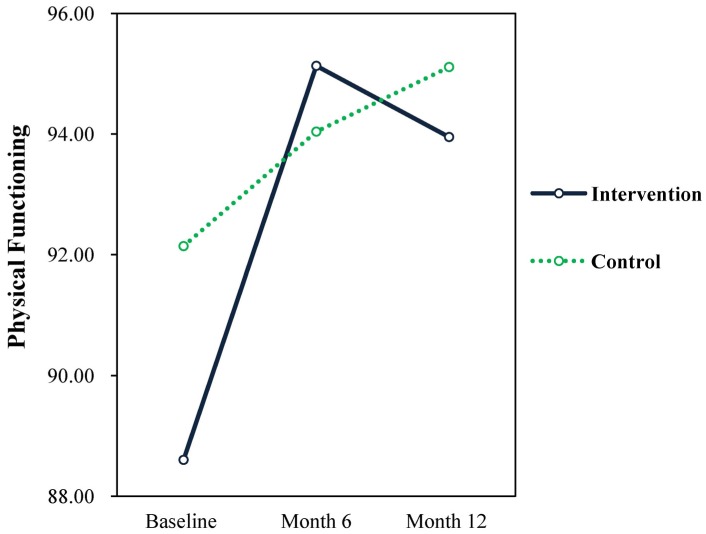
Physical functioning.

Results showed a significant time × treatment interaction effect (F = 6.01, *p* = 0.015, d = 0.344) for physical functioning. The intervention group had significant increases in physical functioning than the control group from baseline to month 6 (See Figure 6). These effects seemed to decline after 6 months. The intervention group scores declined to a level below that of the control. However both groups remained at significantly higher levels on physical functioning from baseline to 12 months. The changes for the control group were significant between baseline and 12 months but this was not the case between baseline and 6 months.

#### 3.5.2. Emotional and Psychosocial Functioning

**Figure 7.**
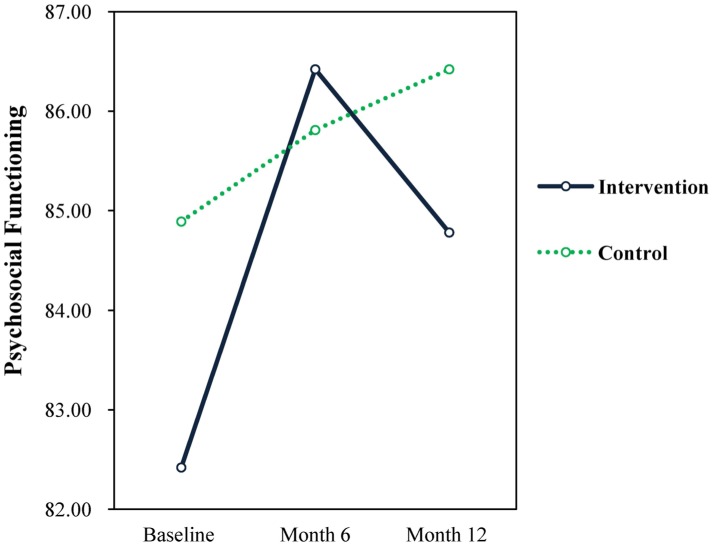
Psychosocial functioning.

For emotional functioning, there were significant time effects for the intervention group from baseline to 6 months (F = 7.88, *p* = 0.006, d = 0.334) and from baseline to month 12 (F = 6.29, *p* = 0.014, d = 0.312). There were significant time effects for psychosocial functioning for the intervention group from baseline to 6 months (F = 10.72, *p* = 0.001, d = 0.411), while the control group remained relatively unchanged over time (see Figure 7).

#### 3.5.3. Social and School Functioning

For school functioning, results from the ANOVA revealed no significant time × treatment interaction and neither were there significant group or time effects. However the means for the experimental group increased between baseline and 6 months even though this change was not significant. There was a significant main effect for social functioning for the experimental group from baseline to 6 months (F = 5.61, *p* = 0.020, d = 0.312), whereas social functioning in the control group remained fairly constant from baseline to 6 months. As seen with the other factors, these effects declined between 6 months and 12 months while the control group experienced significant increases over the same period.

#### 3.5.4. Total Score of HRQL

**Figure 8.**
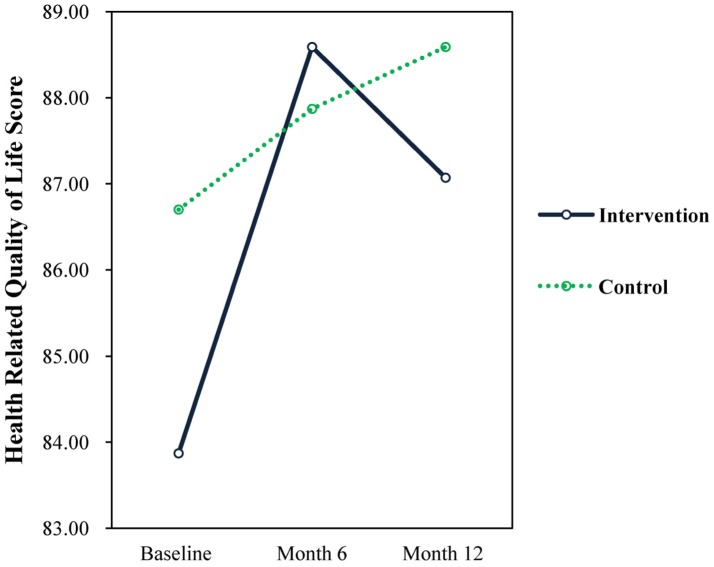
Total HRQL.

Results revealed a significant time × treatment interaction effects for the total score on HRQL (F = 5.93, *p* = 0.016, d = 0.34), indicating that total HRQL score significantly increased from baseline (M = 82.42, SD = 10.96) to 6 months (M = 86.42, SD = 8.33) in the intervention group with minimal improvement in the control group. As observed in the factor scores, the total HRQL score for experimental group dropped below that of the control group from 6 months to 12 months (see Figure 8).

## 4. Discussion

Our study sought to establish if integrating nutrition intervention (culturally tailored food supplement) into antiretroviral treatment (ART) had an impact on physical activity, motor performance and health related quality of life among HIV-positive children in Botswana. Preliminary analyses show that except for social functioning and the total HRQL score, there were no significant differences between the control and experimental group on all the target variables at baseline. The control group had higher scores for social functioning and total HRQL at baseline compared to the experimental group. Overall participants in the two arms were evenly matched.

Our study shows significant improvements in vigorous physical activity for children in both the experimental and the control group between baseline and the 12^th^ month, effectively suggesting the impact of ART and the nutritional intervention. The results are further buttressed by significant improvements in the children's scores on the grip-strength, lower body (legs) power and coordination and the physical functions domain on the HRQL inventory at 12 months. The results show a time by treatment interaction for vigorous physical activity and upper power and coordination with the treatment group showing significant gains over time compared to the control group. It may be that the protein diet made a significant difference in the building of muscle strength and energy balance in the experimental groups. These effects are significant when considered in the context of evidence that suggests that ART, especially protease inhibitors, may have deleterious effects on “peripheral muscle oxygen extraction and utilization during exercise” [29].

Cade and colleagues [29] argue that this may limit capacity to exercise or participate in regular recreational activities. This would have major implications for children's quality of life, overall happiness and wellbeing. The research team in the current study observed that most of the children had difficulty with PA and motor performance tests at baseline but this improved over time. Most performed poorly on the cardiovascular endurance exercise. Functional aerobic impairments have been reported in both adolescents with HIV [30],[31]. This has been attributed to deconditioning resulting from exercise and activity intolerance. It is therefore fair to argue that the combined effects of ART and nutrition led to overall improvements to health and wellbeing which in turn led to increase in PA and performance of motor skills.

Also worth noting are significant improvements in BMI for both groups over time along with decreases in body fat percentage, although the latter was not statistically significant. The children in this study were significantly underweight at baseline. However, it seems they experienced significant gains from ART, nutritional intervention and possible stimulation from the quarterly PA and motor performance assessments. These findings are consistent with results from previous research that examined the relationship between physical activity and quality of life of people living with HIV [30]–[32].

This study shows significant effects for quality of life over time. Specifically, there was significant time by treatment interaction effects for the total HRQL score as well as the psychosocial and physical functioning components of the inventory. These findings are consistent with the findings of a study involving Thai and Cambodian children on ART [8]. The study found a significant relationship between taking ART and improvements in all domains of quality of life except social functioning [8]. HIV infected children reported lower quality of life scores than their non-infected counterparts, suggesting the key role played by ART in improvements to quality of life.

In this study, there were no effects for school functioning for both groups. At baseline, the children in our study reported missing school on many days because they were either bed-ridden or had go and see a doctor. Over time, the children seemed to have fairly good functional ability, as demonstrated by fewer self-reported sick days and hospital admissions. However, even with the health gains they still lagged behind in their school work. Anecdotal reports from parents and caregivers indicated that the children were not doing well academically. Eight of the children had dropped out of school in the year of the study most likely due to ill-health. We recognize that school function, attendance and attrition, as well as academic performance depend on a complex mix of factors. However, we believe our study's findings on school functioning support previous findings that linked low PA, poor nutrition and ill-health to low levels of goal-directed cognitive processes [33],[34]. Evidence suggests that ART alone may not be sufficient and in fact may contribute to neuro-motor decline over time [34]–[36].

An important consideration from these results however is the change in school and social functioning between the groups from baseline to 6 months. The experimental group picked up significantly and caught-up with the control group on these two factors after 6 months even though these effects seem to slow down at 12 months.

A closer examination of group differences in physical functioning, social functioning, emotional functioning, psychosocial functioning and total HRQL shows significant gains for the experimental group at 6 months. Interestingly, the experimental group had lower scores on all these factors at baseline but caught up and in some cases surpassed the control group at 6 months. Once again this suggests a good response to both ART and the high protein micronutrient diet. These effects, however seemed to wear off overtime and in some cases even fell below the control group means at 12 months. It is not clear what could have accounted for this trend. It may be that the novelty of a high protein diet wore off in time. Nonetheless the within-group differences show that both groups had significantly higher scores from baseline to six and 12 months. It has to be borne in mind that a significant number of the children in our study were generally from low-income households and were generally food insecure and protein malnourished at the time of enrollment in the study. Information on the utilization of the food product collected from site visits and on data collection days indicated that the food products were highly consumed.

## 5. Conclusion

Our study has demonstrated a relationship between the integration of nutrition intervention into ARV on physical activity, motor performance, and quality of life among HIV positive. It shows that treatments for children living with chronic diseases like HIV could benefit from dietary interventions. A combination of ART with these factors could enhance the quality of life of children. The results also suggest the possibility of seeing effects increase in interventions lasting more than three months. The strength of our study is in the use of an age and sex matched randomized controlled trial over 12 months. Observing the changes over this period under the level of controls and monitoring built into the protocol gave us more confidence that the effects were due to the intervention.

Our study's limitation is in not doing a PA and motor performance intervention. This was in light of the health condition of the children when they enrolled in the study. However, we still believe the quarterly assessments were generally stimulating and served as a good proxy for improvement to health related quality of life of the children. Overall our findings show that, even with the progress made on the research in this area, more investigations are needed on the associations among wellbeing, physical activity, nutrition, brain health, schooling and academic achievement of children on ART from the world's most affected regions. Studies that compare children with different strains of HIV and those that include comparative samples of non-HIV infected children are likely to be highly informative. Future research that use objective measures of PA and control for confounding variables of HRQL are warranted to better understand the role of an integrated nutrition PA and ART interventions.
